# Biochemical comparison of osteoarthritic knees with and without effusion

**DOI:** 10.1186/1471-2474-12-273

**Published:** 2011-11-28

**Authors:** Nicole M Cattano, Jeffrey B Driban, Easwaran Balasubramanian, Mary F Barbe, Mamta Amin, Michael R Sitler

**Affiliations:** 1 Department of Sports Medicine, West Chester University of Pennsylvania, 855 S. New Street, 313 Sturzebecker HSC, West Chester, PA 19383, USA; 2 Division of Rheumatology, Tufts Medical Center, 800 Washington Street, Box 406, Boston, MA, 02111, USA; 3 Department of Orthopaedic Surgery and Sports Medicine, Temple University, 3509 N. Broad Street, 5th Floor Boyer Pavilion, Philadelphia, PA 19140, USA; 4 Department of Anatomy and Cell Biology, Temple University School of Medicine, 3500 N. Broad Street, Philadelphia, PA 19140, USA; 5 College of Health Professions and Social Work, Temple University, 3307 N. Broad Street, Suite 300, Philadelphia, PA 19140, USA

## Abstract

**Background:**

Several symptom-relieving interventions have been shown to be efficacious among osteoarthritis (OA) patients with knee effusion; however, not every symptomatic knee OA patient has clinical effusion. Results may be over-generalized since it is unclear if effused knees represent a unique pathological condition or subset compared to knees without effusion. The primary purpose of this study was to determine if biochemical differences existed between OA knees with and without effusion.

**Methods:**

The present cross-sectional study consisted of 22 volunteers (11 with knee effusion, 11 without knee effusion) with confirmed late-stage radiographic knee OA (Kellgren-Lawrence score ≥ 3). Synovial fluid samples were collected and analyzed using a custom multiplex enzyme-linked immunosorbent assay to determine eight specific biomarker concentrations (e.g., catabolic, anabolic).

**Results:**

Matrix metalloproteinase (MMP)-3, tissue inhibitor of MMPs (TIMP)-1, TIMP-2, and interleukin-10 were significantly higher in the knees with effusion than in the knees without effusion.

**Conclusions:**

The biochemical differences that existed between knees with and without effusion provide support that OA subsets may exist, characterized by distinct biochemical characteristics and clinical findings (e.g., effusion).

## Background

Osteoarthritis (OA) is a heterogeneous disease disabling more than 27 million United States adults [1]. Patients with knee OA commonly present with pain, stiffness, effusion, and functional impairment. Symptoms may be modulated by effusion as patients with knee effusion are often more symptomatic [2-4] and have greater improvement in pain and function to symptom-modifying interventions than patients without knee effusion [5]. Based on magnetic resonance imaging findings, patients with knee effusion comprise 30 to 76% of patients with knee OA [6,7]. OA knees with effusion, particularly those with active effusion processes, may be a discreet subset of patients with unique clinical characteristics (e.g., effusion, increased symptoms, increased therapeutic response) and at greater risk for cartilage loss over time [8,9]. Although knees with effusion respond well to pain-relieving treatments, there is potential for over generalization of results since it is unclear if OA knees with and without effusion represent unique biochemical conditions. Biochemical analyses may distinguish unique OA phenotypes [10] which may be clinically distinct (e.g., different symptom severity) and respond uniquely to interventions.

The primary purpose of this study was to determine if biochemical differences existed between OA knees with and without clinical effusion. The hypothesis of this study was that OA knees with effusion would have elevated inflammatory mediators when compared to OA knees without effusion. Highly sensitive clinical and biochemical data may help identify discreet subsets of patients that can be targeted for improved diagnosis, interventions, and genotyping of OA, [5,11] ultimately, improving the overall care of knee OA patients.

## Methods

### Study Design and Participants

This cross-sectional study was approved by the Institutional Review Board, and informed consent was obtained from each patient prior to participation in the study. Participants were a sample of convenience and recruited from November 2009 to August 2010 at an orthopedic practice with a board-certified orthopedic surgeon specializing in hip and knee arthroplasty. Twenty-two volunteers (11 knees with effusion, 11 knees without effusion) with late-stage knee OA (Kellgren-Lawrence score ≥ 3) [12] participated in the study. Prior standard weight-bearing anterior-posterior radiographs with knees in full extension were used to assess OA severity utilizing the Kellgren-Lawrence score [13]. New radiographs were ordered and graded by the orthopedic surgeon if the participant's radiographs were taken more than 12 months prior to study inclusion. An investigator-generated medical history form was used to screen for exclusion criteria, as well as to record medication and supplement use, injury history, effusion history, and sport participation history. Patients were excluded from the study if they indicted acute knee trauma (i.e., injury, surgery) within three months of study participation, intraarticular corticosteroid knee injection within thirty days prior to study participation, or documented history of other forms of arthritis (e.g., rheumatoid, gout, pseudogout).

### Effusion Status

Effusion status was based on a clinical examination by the orthopedic surgeon, utilizing observation, palpation, a modified patellar ballottement test [14], and whether or not aspiration of the knee was successful. Knee with effusion was defined as a knee that felt and looked swollen, had a positive patellar ballottement test, and did not require saline injection for synovial fluid aspiration. A knee without effusion was defined as a knee that did not feel or look swollen, had a negative patellar ballottement test, and required a saline injection to successfully aspirate synovial fluid.

### Sample Acquisition, Storage, and Analysis

Upon completion of all study-related documentation and assessment, the participant's involved OA knee joint was aspirated in accordance with the orthopaedic surgeon's standard procedures. The aspiration consisted of vigorously cleaning the needle insertion site with isopropyl alcohol and using an 18 to 21 gauge 3.81 cm disposable needle with a 20 or 60 cm^3 ^syringe to aspirate the joint through the lateral suprapatellar pouch. If the knee did not have an effusion, the physician injected 5 to 15 cm^3 ^of sterile saline into the knee to "washout" the synovial fluid prior to aspiration. A successful saline-assisted aspiration was defined by the recovery of fluid with the color of healthy synovial fluid. For all knees, the physician aspirated as much synovial fluid as could be drawn, which was then visually inspected to ensure that it was a healthy color. Healthy synovial fluid was successfully obtained from all 22 volunteers, and no samples were excluded based on signs of blood, turbidity, or sepsis.

### Synovial Fluid Analysis

A portion (1.0 to 2.0 cc) of the aspirated synovial fluid was placed in a cryovial and stored in a -80°C freezer until all of the samples were collected. Once all synovial fluid samples were collected, they were shipped to Aushon Biosystems Laboratory (Billerica, MA) without centrifuging for analysis. Knee synovial fluid biomarker concentrations were analyzed with a custom human SearchLight Proteome Multiplex Protein Array. The custom array is a quantitative multiplexed sandwich enzyme-linked immunosorbent assay, containing eight different antibodies. The study focused on eight specific biomarkers, which were categorized as follows: (1) protective or anabolic-associated biomarkers (i.e., interleukin [IL]-10, IL-13, tissue inhibitor of matrix metalloproteinase [TIMP]-1, TIMP-2, and osteoprotegerin [OPG]), (2) anabolic biomarker associated with cartilage ossification and neovascularization (i.e., vascular endothelial growth factor [VEGF]), (3) catabolic-associated biomarker (i.e., matrix metalloproteinase [MMP]-3), and (4) pro-inflammatory associated biomarker (i.e., IL-1β). All of these biomarkers have been reported to be altered in the synovial fluid of OA joints in comparison to healthy, non-OA knees [10,15]; however, no distinction was made between OA knees with and without effusion in these previous studies.

Concentrations of each biomarker were analyzed in duplicate and then averaged. In order to take into account the injection of saline into the non-effused knees, cytokine data were normalized to the total protein content of each sample, which was determined by a bicinchoninic acid assay (ThermoScientific, Chicago, IL). Normalization of the data allowed for comparison between the two groups, as knees without effusion required saline-assisted aspirations that diluted an unknown amount of synovial fluid, which was not the case for the knees with effusion.

Validity of the SearchLight proteome assays has been established through the spiked recovery rates for the 8 biomarkers analyzed, which ranged from 62 to 134% (mean recovery ranges from 76 to 114%). Sensitivity and intra-assay coefficient of variation measures for the eight biomarkers analyzed were: IL- 10 (sensitivity = 0.8 pg/mL, intra-assay coefficient of variation = 6.7%), IL-13 (sensitivity = 0.4 pg/mL, intra-assay coefficient of variation = 8.1%), TIMP-1 (sensitivity = 9.8 pg/mL, intra-assay coefficient of variation = 13.8%), TIMP-2 (sensitivity = 9.8 pg/mL, intra-assay coefficient of variation = 8.4%), OPG (sensitivity = 0.4 pg/mL, intra-assay coefficient of variation = 8.9%), VEGF (sensitivity = 9.8 pg/mL, intra-assay coefficient of variation = 4.8%), MMP-3 (sensitivity = 19.6 pg/mL, intra-assay coefficient of variation = 8.9%), and IL-1β (sensitivity = 0.4 pg/mL, intra-assay coefficient of variation = 8.2%).

### Statistical Analyses

Data were analyzed using descriptive and inferential statistics with SPSS for Windows Version 18.0 (SPSS Inc., Chicago, IL) and GraphPad Prism (GraphPad Software, Inc., La Jolla, CA) statistical programs. Data were evaluated for normal distributions by evaluating for skewness and kurtosis. Because synovial fluid biomarker concentrations were not normally distributed, Mann Whitney U tests were used to determine if significant differences existed between the median values of two groups. A two-way analysis of variance was used to determine if differences in anti-inflammatory medication use affected biomarker results. The alpha level (*p *≤ 0.05) was adjusted with Bonferroni corrections for the 8 biomarker comparisons (*p *≤ 0.006).

## Results

### Participant characteristics

Osteoarthritis (OA) was confirmed in each group radiographically; Kellgren-Lawrence scores are reported in Table 1. The knees with effusion group consisted of 6 females and 5 males (mean age of 66.1 ± 9.4 years and mean body mass index of 33.61 ± 6.40 kg/m^2^; nine with Kellgren-Lawrence scores of 3 and two with Kellgren-Lawrence scores of 4). The knees without effusion group consisted of 7 females and 4 males (mean age of 62.9 ± 5.3 years and mean body mass index of 34.26 ± 7.68 kg/m^2^; nine with Kellgren-Lawrence scores of 3 and two with Kellgren-Lawrence scores of 4). Age and body mass index were not significantly different between the two groups. Three participants in the knees without effusion group self-reported no history of knee swelling. The remaining eight participants in the knees without effusion group were currently having knee discomfort and were not currently effused however, each reported a history of knee swelling. The knees with effusion group had 10 of 11 participants and the knees without effusion group had 8 of 11 participants taking an anti-inflammatory medicine for management of their OA symptoms (Table 1).

**Table 1:** Patient characteristics and medication use

Participant	Gender	KL Grade	Age	BMI	NSAID	Medication
Effusion group						
1	Male	3	62	49.61	No	None
2	Female	4	67	34.64	No	Acetaminophen, calcium
3	Female	3	59	40.02	Yes	Ibuprofen
4	Female	3	55	30.73	Yes	Naproxen
5	Female	3	64	34.92	Yes	Ibuprofen
6	Female	3	56	28.79	Yes	Ibuprofen
7	Male	3	74	28.95	Yes	Ibuprofen, Acetaminophen
8	Male	3	85	28.25	Yes	Naproxen
9	Female	3	77	30.47	Yes	Ibuprofen
10	Male	4	68	29.33	Yes	Ibuprofen
11	Male	3	60	34.02	Yes	Naproxen, Glucosamine

No effusion group						
12	Female	3	65	36.07	No	Acetaminophen
13	Male	3	69	35.05	No	Acetaminophen, Calcium
14	Male	4	66	41.23	No	Acetaminophen
15	Female	3	56	31.06	No	Acetaminophen
16	Female	3	70	31.65	Yes	Naproxen
17	Female	3	69	23.61	Yes	Chondroitin, Ibuprofen
18	Male	3	64	33.54	Yes	Ibuprofen
19	Male	3	59	29.11	Yes	Ibuprofen
20	Female	3	59	39.14	Yes	Naproxen
21	Female	4	57	50.95	Yes	Ibuprofen
22	Female	3	58	25.46	Yes	Acetaminophen, Ibuprofen

Note: KL = Kellgren Lawrence, BMI = Body Mass Index, NSAID = Nonsteroidal Anti-inflammatory Drug

### Biochemical distinction of knees with and without effusion

Median and confidence intervals for synovial fluid concentrations are presented in Table 2. The knees with effusion group had significantly higher IL-10 (217%), TIMP-1 (9913%), and TIMP-2 (1213%) median concentrations (protective biomarkers) than the knees without effusion group. The knees with effusion group also had significantly higher median MMP-3 concentrations (62204%; catabolic biomarker) than the knees without effusion group. No other synovial fluid biomarker concentrations were statistically different. No significant interaction (p = 0.322) was found between anti-inflammatory medication use and biomarker results when a two-way analysis of variance was conducted.

**Table 2:** Synovial fluid biomarker concentrations (pg/μg of total protein) of osteoarthritic knees with and without effusion

	Non-Effused (n = 11)	Effused (n = 11)		
Biomarker	Median (95% CI)	Median (95% CI)	*p *value	*r*
IL-1β	0.000009 (0.000003 - 0.000023)	0.000012 (0.000006 - 0.000020)	0.714	-0.078
IL-10	0.000017 (0.000007 - 0.000039)	0.000054 (0.000037 - 0.000184)	0.003*	-0.625
IL-13	0.000057 (0.000038 - 0.000173)	0.000047 (0.000034 - 0.000087)	0.670	-0.091
TIMP-1	0.519388 (0.000000 - 13.457844)	52.008696 (38.851239 - 80.618044)	< 0.001*	-0.791
TIMP-2	0.340405 (0.000000 - 2.521999)	4.471101 (3.758427 - 5.662756)	0.002*	-0.665
MMP-3	0.130385 (0.000000 - 6.931912)	81.236149 (0.000000 - 334.057639)	< 0.001*	-0.819
OPG	0.000148 (0.000045 - 0.000281)	0.000104 (0.000116 - 0.000447)	0.224	-0.259
VEGF	0.001912 (0.000000 - 0.020470)	0.019389 (0.011830 - 0.033989)	0.013	-0.515

Note: CI = confidence interval, r = effect size, IL = interleukin, TIMP = tissue inhibitor of metalloproteinase, MMP = matrix metalloproteinase, OPG = osteoprotegerin, and VEGF = vascular endothelial growth factor. * *p *≤ 0.006 was considered significant.

## Discussion

OA knees with effusion were biochemically distinct from OA knees without effusion, providing support for the hypothesis that effusion status may define an OA phenotype [11]. OA knees with effusion had elevated levels of protective biomarkers (IL-10, TIMP-1, TIMP-2) and the catabolic-associated biomarker MMP-3 compared to knees without effusion. The current study is the first to compare the biochemical profiles between OA knees with and without effusion.

Three of the elevated biomarkers (TIMP-1, TIMP-2, and MMP-3) in the OA knees with effusion group are directly involved in the regulation of articular cartilage extracellular matrix turnover [16,17]. MMP-3 is elevated in nearly all diarthrodial joint OA tissues [18]. MMP-3 promotes proteoglycan breakdown and is expressed in a stage-dependent manner: elevated higher in early-stage OA than in late-stage OA [18]. TIMP-1 and TIMP-2, inhibitors of MMPs, have also been reported to be elevated in knee OA [16]. The increase in protective biomarkers may represent a failing attempt to restore homeostasis as the joint degenerates [19]. The current study controlled for stage-dependent biochemical changes by analyzing late-stage OA only (Kellgren-Lawrence score ≥ 3). The findings of the current study suggest that the OA knees with effusion had greater extracellular matrix turnover than the knees without effusion group, at least at the time of synovial fluid collection. The detected changes are indicative of active cellular processes at the time of fluid collection since eight of the eleven participants in the knees without effusion group had prior history of knee effusion and biomarkers have short life spans. However, it remains unclear if the increased extracellular matrix turnover caused the effusion or if the presence of effusion lead to increased extracellular matrix turnover. Future studies should focus longitudinally on effusion status, biochemical changes, and the progression of changes in function and structure to determine causality.

Anabolic and protective activity increases when there is structural damage to the articular cartilage (e.g., macrotrauma, microtrauma) in an attempt to repair damaged articular cartilage [20]. Collagen synthesis increases significantly in late-stage OA but is insufficient to promote recovery [21]. The elevated protective biomarkers (i.e., IL-10, TIMP-1, TIMP-2), as well as the increase in MMP-3, in the knees with effusion group may suggest that these knees were in a more active degenerative state compared to the knees without effusion group. In a limited sample of knees (*n *= 20) with minimal baseline cartilage damage, the presence of synovitis or effusion led to a trend for increased odds of rapid cartilage loss [8,9]. The knees with effusion group may represent a unique subset of OA but more research is needed to further define the subset characteristics (e.g., rate of disease progression). Additionally, further studies on biomarkers specific to collagen turnover may help to validate that knees with effusion may be an OA subset that are in a more active cartilage degenerative state than knees without effusion.

Joint effusion is associated not only with cartilage turnover but also with synovitis. The use of imaging along with biomarkers could help to determine the level of effusion associated with synovitis. Synovitis is common in OA and not just to the end stage of the disease process, as some degree of synovitis is present in early OA as well [22]. A total of 57% of knees with synovitis are associated with elevated concentrations of the inflammatory biomarker serum C-reactive protein [23]. The presence of synovitis does not always result in an increase in inflammatory mediators. The OA knee without effusion could also be generating inflammatory mediators. Based on the current study, it is likely that synovitis with elevated inflammation does not vary between groups, although IL-10 was elevated in the knees with effusion group compared to the knees without effusion group. It is unclear which biochemical mediators stimulate IL-10, as IL-10 is elevated in OA joints and associated with chondroprotective as well as anti-inflammatory roles. In the temporomandibular joint, a biochemical comparison of joints with and without effusion demonstrated significant differences in the concentrations of soluble cytokine receptors but IL-1 receptor antagonist was not statistically different [24]. The implications to the knee are yet to be determined, as it is unknown if differences exist between different synovial joints.

In the current study, medication and supplement use were controlled for by having patients self-report their use and then analyzing if there were any significant biochemical differences due to that use. Despite the disparity in anti-inflammatory use between the two groups, none of the inflammatory markers were statistically different between groups. Accordingly, anti-inflammatory medication use had no overall effect on biomarker concentrations. Patients may need to consume anti-inflammatory medication regularly for the medications to influence biomarker concentrations. It is plausible that anti-inflammatory medication use did not affect biomarker concentrations because they were not consumed consistently, but the study questionnaires were not designed to confirm consistent medication use. Future research should be conducted on patients using a broad range of inflammatory mediators and more detailed medication use data to determine the extent that medication use effects biochemical differences in knees with and without effusion.

The heterogeneity of OA is hindering progress in developing new interventions and determining the effectiveness of existing interventions for OA. The concept of phenotyping subsets of OA patients has begun to gain momentum [11,25,26], but which phenotypes are clinically relevant remains unclear. More research is needed to explore the biochemical differences between an interaction of effusion status and symptoms (e.g., moderate-high symptoms with effusion, low symptoms with effusion, low symptoms with non-effusion). This may permit regression modeling to determine the association between clinical variables (e.g., quantification of effusion, symptoms, and biochemical mediators) and the development of individualized scores (continuous gradient model), taking one step forward in stratifying subsets/phenotypes [11].

OA stratifications may be dependent on stable variables (e.g., mechanism of onset, genetic risk factors, involved joints), transient variables (e.g., effusion), or their interaction. Effusion status is commonly conceptualized as a transient stratification; however, there may be a stable stratification based on the history of knee effusion (e.g., no history of knee effusion, history of knee effusion). In the current study, three participants within the knees without effusion group had no self-reported history of effusion. These participants trended to having lower biomarker concentrations than the other eight participants within the knees without effusion group who self-reported a history of effusion. The three participants with no self-reported history of effusion were also three of the five knees with the lowest concentrations of TIMP-1, TIMP-2, MMP-3, and VEGF. Based on these findings, further research should be conducted to determine if history of effusion could possibly be used to determine stable stratifications or phenotypes of OA. This may have implications when assessing the effectiveness of interventions through clinical trials. Each biochemical subset (stable or transient) may respond differently to interventions. Future research should focus on the effects of interventions on the biochemical level in an effort to improve the overall clinical care of OA.

This study was limited by the cross-sectional design and the relatively small sample size. Future studies would be strengthened by increasing the sample size, as well as longitudinally following the patients. Furthermore, the lack of imaging modalities (e.g., magnetic resonance imaging, ultrasound) to objectively measure effusion and synovitis levels limits the generalizability of the results. Even with these limitations this study detected biochemical differences between knees with and without effusion that warrants further research.

## Conclusions

The biochemical differences that existed between knees with and without effusion provide preliminary data based on a relatively small sample size to support that OA subsets may exist, characterized by distinct biochemical characteristics and clinical findings (e.g., effusion). These current data suggest that this area of research warrants more attention in the future. Larger cohort studies should be conducted to verify this finding. Presumably, the biochemical distinction suggests that OA knees with effusion may need to be managed differently than OA knees without effusion.

## Lists of abbreviations

OA: osteoarthritis; MMP: matrix metalloproteinase; TIMP: tissue inhibitor of MMPs; IL: interleukin; OPG: osteoprotegerin; VEGF: vascular endothelial growth factor.

## Competing interests

The authors declare that they have no competing interests.

## Authors' contributions

All authors read and approved the final manuscript. NMC participated in the design of the study, obtained funding, performed data collection, analyses, and drafted the manuscript. JBD conceived and designed the study, helped with data interpretation, and critical revision of the manuscript for important intellectual content. EB participated in the design of the study, performed aspirations, and diagnosed radiographs. MFB participated in the design of the study, helped with data interpretation, and critical revision of the manuscript for important intellectual content. MA performed analyses for protein normalization and sample shipping. MRS participated in the design of the study, helped with data interpretation, and critical revision of the manuscript for important intellectual content.

## Acknowledgements

This research was funded and supported by West Chester University Faculty Development Grant to NMC for supplies and sample analyses.

## Pre-publication history

The pre-publication history for this paper can be accessed here:

http://www.biomedcentral.com/1471-2474/12/273/prepub
